# A Condition-Monitoring Method for Rolling Bearings Based on Dynamic Asynchronous Peak-Factor Ratios

**DOI:** 10.3390/s23218939

**Published:** 2023-11-02

**Authors:** Guanhua Zhu, Quansi Huang, Zeyu Zhang

**Affiliations:** 1 Guangdong Provincial Key Laboratory of Petrochemical Equipment and Fault Diagnosis, Guangdong University of Petrochemical Technology, Maoming 525000, China; zhugh@gdupt.edu.cn; 2 School of Automation, Guangdong University of Petrochemical Technology, Maoming 525000, China; 3 College of Informatics, Huazhong Agricultural University, Wuhan 430070, China; zhangzeyu@mail.hzau.edu.cn

**Keywords:** rolling bearings, early failure, condition monitoring, Honey Badger Algorithm, dynamic asynchronous peak factor ratio, envelope spectrum

## Abstract

In response to issues such as the lack of capability for timely early warning and the difficulty in monitoring the status of rolling bearings, a condition-monitoring method for rolling bearings based on the Honey Badger Algorithm (HBA) for optimizing dynamic asynchronous periods is proposed. This method is founded on the peak factor and involves comparing peak factors at different periods to construct a dynamic asynchronous peak-factor-ratio-monitoring index, which is then optimized using the HBA. Simulated experiments were carried out using the XJTU-SY dataset. The results indicate that, compared to the early warning times defined by international standards, the warning times provided using this method are consistently over 33 min in advance within the test dataset. Additionally, an envelope spectrum analysis of the warning data confirms the existence of early faults. This demonstrates that the monitoring indicator developed in this paper is capable of delivering earlier and more accurate early fault warnings and condition monitoring for rolling bearings.

## 1. Introduction

Rolling bearings, which are crucial components of rotating machinery, can be influenced by various loads, vibrations, and environmental conditions during their operation. The performance of bearings deteriorates over time, gradually transitioning from a normal state to a failure state. Therefore, the timely and accurate detection of potential faults in rolling bearings is essential to ensure stable operation and prolong the lifespan of mechanical equipment [1]. Today, there are several methods available for bearing monitoring, such as temperature analysis, oil analysis, noise analysis, current and voltage analysis, radio frequency analysis (RF), acoustic emission monitoring, and vibration analysis [2,3,4]. Among these methods, time-domain vibration signals are the most fundamental and direct signals in the monitoring of rolling bearings. They also contain valuable information about fault characteristics [5]. Detecting fault characteristics directly from vibration signals for bearing condition monitoring would be highly beneficial [6,7].

In vibration feature analysis, the probability density function of vibration signals can better reflect fault information. From the probability density function of vibration signals, dimensional indicators (such as mean and root mean square) and dimensionless indicators (such as waveform factor, peak factor, impulse factor, and margin factor) can be derived [8,9,10]. In practice, while dimensional indicators are sensitive to bearing fault characteristics and increase as faults develop, they can be easily disturbed by changing operating conditions such as load and speed [11], making them less stable and affected by performance. To address these limitations of dimensional indicators in the monitoring of bearing conditions, researchers designed dimensionless indicators by taking the ratio of two-dimensional indicators. These dimensionless indicators are less sensitive to disturbances in vibration monitoring signals and exhibit more stable performance. Notably, these dimensionless indicators are less affected by changes in signal amplitude and frequency, making them less dependent on machine operating conditions. Therefore, dimensionless indicators have been widely applied in the diagnosis of rotating machinery faults [8,12,13]. Among dimensionless indicators, amplitude factor and impulse factor are particularly sensitive to bearing impacts, especially in early-stage faults, where these factors increase rapidly compared to other dimensionless indicators. However, they still exhibit significant fluctuations [8].

For over three decades, the condition monitoring of rolling bearings has been a subject of research interest. In practice, bearings are typically maintained at fixed time intervals. However, the performance of bearings does not change linearly due to the influence of environmental and operational conditions. Therefore, the online monitoring of bearings is necessary to achieve higher efficiency. Methods based on condition monitoring are employed to enhance bearing performance, efficiency, lifespan, and productivity while reducing internal and external damage. The monitoring and fault detection of bearings have become crucial to prevent unexpected failures and minimize unplanned downtime. In recent years, methods for the monitoring of bearing conditions based on signal processing and data mining have become mainstream [14]. Xiong J. et al. [8] proposed a data-fusion method based on dimensionless indicators, which calculates five dimensionless indicators from real-time collected raw data and then uses support vector machines for fault-type projection to address the problem of low fault diagnosis accuracy due to the imperfections of old dimensionless indicators. Qiu et al. [14,15] employed Morlet wavelet transform for denoising bearing vibration signals, followed by self-organizing maps for health assessment of abnormal bearings, ultimately evaluating the current condition of the bearings. Qiao Z. and Lei Y. [1,16], considering that fault features of initial mechanical faults are difficult to detect, used random resonance and improved random resonance to enhance and extract weak fault features in vibration signals. Antoni and Randall [17,18] introduced the spectral kurtosis indicator to characterize bearing fault signals. The spectral kurtosis indicator detects the presence of bearing faults by examining abnormally high kurtosis values in specific frequency bands. The spectral kurtosis indicator is associated with pulse repetition rate, sampling rate, and pulse intensity. Qin H. et al. [19] proposed an effective fault diagnosis method using multiscale dimensionless indicators (MSDI) and random forests, primarily addressing the low fault-diagnosis accuracy of traditional dimensionless indicators for nonlinear, non-stationary dynamic signals in rotating machinery. This method employs a variational mode decomposition on vibration signals, constructs six types of MSDIs based on the decomposed signals, and uses the Fisher criterion to select the top MSDIs as inputs for classification in random forests. Yang C. et al. [20] proposed a digital-twin-driven composite fault-diagnosis method that combines real and virtual data. This method aims to ensure the safe production of underwater systems. It involves real-time monitoring for safety and establishes a digital twin model by incorporating the Bernoulli equation along with loss, control, and state parameters. In the case of a single fault, the digital twin model verifies the results of fault diagnosis models and corrects any errors. However, in the event of composite faults, it is diagnosed by combining virtual data from the digital twin model with real data from the physical system. Civera M. et al. [21] proposed a state-monitoring method that uses instantaneous spectral entropy and continuous wavelet transform to address the frequent failures associated with aging in wind turbines. This method involves anomaly detection and fault diagnosis from historical vibration data of the gearbox.

Currently, the application of non-dimensional indicators for the condition monitoring of rolling bearings is limited, and traditional non-dimensional indicators have the drawback of significant fluctuations during monitoring. Therefore, addressing the issues mentioned above, this paper proposes a method for the condition monitoring of rolling bearing based on the Dynamic Asynchronous Amplitude Factor Ratio. The Honey Badger Algorithm (HBA) [22] is a new metaheuristic algorithm proposed in 2021, mainly simulating the digging behavior of honey badgers and their dynamic search behavior for honey. HBA offers advantages such as its simplicity of structure, ease of implementation, and good optimization performance when dealing with various types of optimization problems [23]. In this paper, we use HBA to optimize the short average period, offset period, and long average period in dynamic asynchronous peak factor ratios. The main contributions of this paper are as follows.

1. The paper introduces a novel monitoring indicator, the dynamic asynchronous peak factor ratio. This application broadens the field of the condition monitoring of rolling bearings, providing a new monitoring metric for early fault warnings and status monitoring of rolling bearings.
2. By employing the HBA algorithm, the paper optimizes the critical parameters of the monitoring indicator. This step enhances the efficiency and accuracy of the monitoring method, ensuring more timely and reliable early fault warnings and status monitoring.
3. Through practical experiments, the effectiveness of the monitoring method proposed in this paper has been substantiated. The experimental results indicate that, in comparison to the early warning times specified by international standards, this method provides warnings at least 33 min in advance. Additionally, by subjecting the warning data to envelope spectrum analysis, the presence of early faults has been confirmed, thereby enhancing the ability to detect early faults in rolling bearings.

The structure of this paper is as follows. Section 2 reviews the dynamic asynchronous peak factor ratio monitoring indicator proposed in this paper, the principles of the Honey Badger Algorithm, and envelope spectrum analysis. Section 3 conducts simulation experiments and analyzes the experimental results of the method proposed in this paper using the XJTU-SY dataset. Finally, Section 4 conclusions and future work.

## 2. Proposed Bearing Condition Monitoring Method

By utilizing the new dimensionless metric introduced in this paper, the Dynamic Asynchronous Peak Factor Ratio, we monitor early-stage faults in bearings. Addressing the challenge of determining parameter combinations within the Dynamic Asynchronous Peak Factor Ratio, we propose employing HBA for optimization, allowing it to automatically search for the optimal parameters. Using the optimized parameters, we monitor bearings with the Dynamic Asynchronous Peak Factor Ratio, obtaining the warning time. To verify the presence of early-stage faults in the bearings at the warning time, we perform envelope spectrum analysis on the data collected during the warning period.

### 2.1. Dynamic Asynchronous Peak Factor Ratio

The Dynamic Asynchronous Amplitude Factor Ratio is grounded in two identical non-dimensional indicators known as amplitude factors. The method entails the individual averaging of these non-dimensional indicators across short, offset, and long periods, followed by their division. By employing diverse period averages on two identical indicators, a fresh non-dimensional indicator is formulated. Dynamic asynchrony is characterized as the ratio between two non-dimensional indicators sharing the same dimensions but varying periods. The expression for dynamic asynchrony is presented below:

(1) $$F\left(T_{s},T_{o},T_{l}\right)=\frac{f(T_{s})}{f(T_{o},T_{l})}$$

where $T_{s}$, $T_{o}$, $T_{l}$, $f(T_{s})$, $f(T_{o},T_{l})$, and $F(T_{s},T_{o},T_{l})$ represent the short-term averaging period for the numerator dimensionless indicator, the offset period for the denominator dimensionless indicator, the long-term averaging period for the denominator dimensionless indicator, the dimensionless indicator obtained by averaging over short periods, the dimensionless indicator obtained by offsetting and then averaging over long periods, and the resulting dynamic asynchronous dimensionless indicator, respectively.

Since the peak factor is not influenced by factors such as bearing size or rotational speed or monitoring the trend of peak factor values over time, it provides timely early warnings for rolling bearings. Therefore, the dimensionless indicator used in this method is the peak factor. The peak factor is defined as the ratio of the peak value to the root mean square value, and its expression is as follows:

(2) $$f\left(C\right)=\frac{\max(|x\left(i\right)|)}{\sqrt{\frac{1}{N}\overset{N}{\underset{n=1}{\sum }}{\left(x(i)\right)}^{2}}}$$

where x(i), $\max(\left|x(i)\right|)$, and $\sqrt{\frac{1}{N}\overset{N}{\underset{n=1}{\sum }}{\left(x(i)\right)}^{2}}$, the denominator, represent the time-series data with i=1∼N data points, the maximum value among *n* absolute value data points, and the root mean square value of *n* data points, respectively.

In summary, this study introduces a novel Dynamic Asynchronous Peak Factor Ratio by comparing two peak factors with different periods, which serves as a monitoring indicator for the early fault detection of bearings.

Compared to traditional dimensional and dimensionless monitoring indicators, the advantages of the monitoring indicator proposed in this paper are as follows:

1. By performing calculations at different intervals, it can suppress interference caused by human factors or occasional shocks, thereby enhancing the early fault-detection capabilities of rolling bearings.
2. Due to its dimensionless property, the dynamic asynchronous peak factor ratio is theoretically unaffected by factors such as bearing size and rotational speed, which gives it good stability in various environmental conditions. Moreover, the process of constructing and applying this indicator is not overly complex.
3. The dynamic asynchronous peak factor ratio can more accurately identify early faults in rolling bearings, thereby facilitating the implementation of appropriate maintenance measures before the issue deteriorates further.

An early fault warning model for bearings based on the dynamic asynchronous peak factor ratio is established, with key parameters including the short-term averaging period ($T_{s}$), the offset period ($T_{o}$), and the long-term averaging period ($T_{l}$). Generally, a larger $T_{s}$ results in smaller peak amplitudes in the indicator, potentially leading to delayed warnings in the model and inadequate coverage of early bearing faults. The offset period, $T_{o}$, fine-tunes the starting point for long-term averaging and is ideally set between the minimum value of $T_{s}$ and the maximum value of $T_{l}$. A larger $T_{l}$ can effectively suppress waveform spikes caused by occasional impacts during machine operation, thus enhancing the model’s ability to detect real machine faults, particularly early faults, and deliver timely and precise warnings. Additionally, $T_{l}$ should numerically surpass $T_{s}$. The optimization ranges for these three parameters are detailed in Table 1 for reference. Given that the parameters in the dynamic asynchronous peak factor ratio play a pivotal role in enhancing its early bearing fault warning capabilities, precisely defining the values of $T_{s}$, $T_{o}$, and $T_{l}$ to optimize the early bearing fault warning model can be a complex endeavor. To achieve accurate parameter determination, this study introduces the HBA as an effective method for optimizing the parameters within the early bearing fault warning model.

### 2.2. HBA Principles

In this article, we will provide a brief overview of the HBA; for a more detailed analysis, please refer to [22]. The HBA mimics the foraging behavior of honey badgers. Honey badgers, to find a food source, either dig using their sense of smell or follow guide birds. The phase where they dig using their sense of smell is referred to as the digging mode, while the phase where they follow guide birds is called the honey mode.

In the HBA algorithm, the overall representation of candidate solutions is given by

(3) $$\left(\begin{array}{ccccc} x_{11} & x_{12} & x_{13} & \cdots & x_{1j}\\ x_{21} & x_{22} & x_{23} & \cdots & x_{2j}\\ \vdots & \vdots & \vdots & \ddots & \vdots \\ x_{i1} & x_{i2} & x_{i3} & \cdots & x_{ij} \end{array}\right)$$

Here, the position of the *i*th honey badger can be represented as $\left[x_{i1},x_{i2},\cdots ,x_{ij}\right]$.

Step 1: Initialize the positions of honey badgers. The expression is as follows:

(4) $$x_{i}=lb_{j}+r_{1}\times \left(ub_{j}-lb_{j}\right)$$

where $x_{i}$ is the position parameter of the *i*th honey badger. $lb_{j}$ is the lower limit of the search range for the *j*th parameter. $ub_{j}$ is the upper limit of the search range for the *j*th parameter. $r_{1}$ is a random number between 0 and 1.

Step 2: The speed of honey badgers’ movement primarily depends on the magnitude of intensity $I_{i}$, which is defined using Equation (5). If $I_{i}$ is stronger, honey badgers will move very quickly, and vice versa.

(5) $$\begin{array}{cc} I_{i} & =r_{2}\times \frac{S}{4\pi d_{i}^{2}}\\ S & ={\left(x_{i}-x_{i+1}\right)}^{2}\\ d_{i} & =x_{prey}-x_{i} \end{array}$$

where *S* represents the source strength or concentration intensity, while $d_{i}$ signifies the distance between the hive and the *i*th honey badger. $x_{prey}$ denotes the location of the prey, which corresponds to the position of the optimal honey badger in the HBA. Additionally, $r_{2}$ is a random number ranging between 0 and 1.

Step 3: The parameter α, known as the density factor, regulates the temporal variation in randomness during foraging, ensuring a seamless transition from exploration to exploitation. Its mathematical expression is as follows:

(6) $$\alpha =C\cdot e^{\left(\frac{-t}{t_{\max}}\right)}$$

where C=2. $t_{\max}$ is the maximum number of iterations.

Step 4: The honey badger position is updated through two phases: the digging mode and the honey mode. The selection between these modes is determined by a random number, denoted as *r*. Here, *r* is a random value ranging from 0 to 1. If r≤0.5, the digging mode is chosen for updating the honey badger position; otherwise, the honey mode is selected to update its position.

Step 5: The honey badger’s digging action resembles a heartbeat-like pattern, and its motion equation is expressed as follows:

(7) $$\begin{array}{cc} x_{new}= & x_{prey}+F\times \beta \times I_{i}\times x_{prey}+F\times r_{3}\\ \; & \times \alpha \times d_{i}\times \left|\cos\left(2\pi r_{4}\right)\times \left[1-\cos(2\pi r_{5})\right]\right| \end{array}$$

where β, which is set to 6 by default, represents the honey badger’s food-acquisition ability. $r_{3}$, $r_{4}$, and $r_{5}$ are random numbers ranging from 0 to 1. Additionally, the parameter *F* plays a pivotal role in dictating HBA search direction, and its mathematical representation is as follows:

(8) $$F=\left\{\begin{array}{cc} 1 & if\;r_{6}\leq 0.5\\ -1 & else \end{array}\right.$$

where $r_{6}$ is a random number between 0 and 1.

Step 6: The expression for updating positions during the honey mode is:

(9) $$x_{new}=x_{prey}+F\times r_{7}\times \alpha \times d_{i}$$

where $x_{new}$ refers to the new position of the honey badger. $r_{7}$ is a random number between 0 and 1.

**Figure 1:**
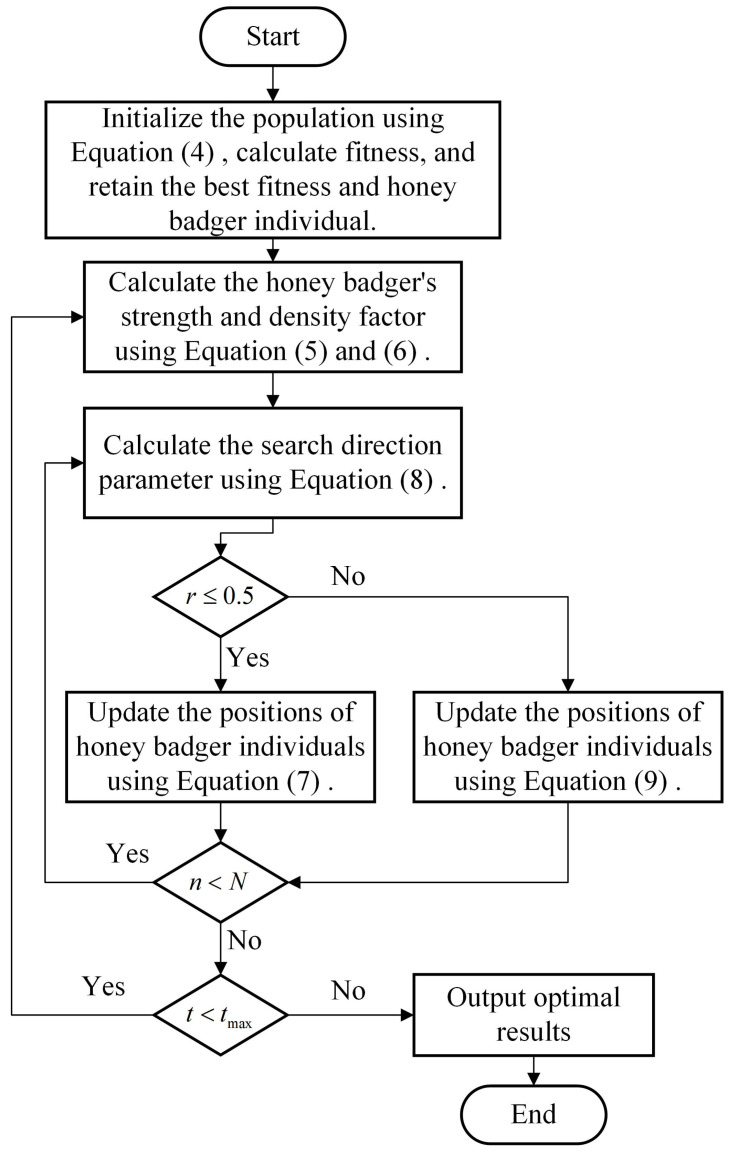
Flowchart of the HBA approach.

In summary, this paper applies vibration data within the context of the HBA algorithm. Subsequently, HBA is utilized to optimize the parameters $T_{s}$, $T_{o}$, and $T_{l}$. The parameter ranges subjected to optimization by HBA are detailed in Table 1, while the specific optimization process of HBA is illustrated in Figure 1.

### 2.3. Envelope Spectrum Analysis

The Envelope Spectrum is a demodulation technique used for signal analysis, and it is commonly applied in the assessment of vibrations and pulse signals within bearing-related applications. In such scenarios, these signals frequently undergo modulation due to inherent vibrations. The envelope spectrum plays a pivotal role in demodulating and extracting these low-frequency impact signals, thereby facilitating fault detection and diagnosis [24]. First, it is necessary to subject the processed signal to appropriate bandpass filtering to enhance the bearing fault frequencies. Performing the Hilbert transform on the filtered signal yields its analytic signal conjugate part.

(10) $$H\left[x\left(t\right)\right]=\frac{1}{\pi }\int_{-\infty }^{\infty }\frac{c(\tau )}{t-\tau }d\tau$$

Therefore, the analytic signal z(t) can be represented as

(11) $$z\left(t\right)=x\left(t\right)+j\bar{H[x(t)]}$$

It can also be expressed as

(12) $$z\left(t\right)=r\left(t\right)\cdot e^{j\theta (t)}$$

where x(t) is the filtered vibration signal, $\bar{x(t)}$ is the Hilbert transform of the filtered vibration signal, z(t) is the analytic signal, and $r\left(t\right)=\sqrt{x{\left(t\right)}^{2}+\bar{x{\left(t\right)}^{2}}}$.

Then, we perform a Fourier transform on r(t) to obtain an envelope spectrum capable of identifying bearing fault characteristics. Subsequently, we calculate the fault’s characteristic frequency. Since this paper primarily focuses on predicting outer race bearing faults, the characteristic frequency for outer race faults is as follows:

(13) $$f_{o}=\frac{z}{2}\left(1-\frac{d}{D}cos\alpha \right)f_{r}$$

where *D* is the diameter of the rolling elements, *d* is the bore diameter of the bearing, α is the contact angle of the rolling element bearing, *N* is the number of rolling elements, and $f_{r}$ is the inner raceway of rotational frequency.

Finally, we observe the envelope spectrum chart, and if there is a peak near the characteristic frequency of outer race faults, it can be determined as an outer race fault.

## 3. Experimental Results and Discussion

To validate the effectiveness and accuracy of the Dynamic Asynchronous Peak Factor Ratio monitoring indicator proposed in this paper for early fault warning, three experiments were conducted on the XJTU-SY dataset.

1. The first experiment was parameter sensitivity analysis, showing how warning times change with variations in parameters if they are not optimized.
2. The second experiment was about optimizing the parameters of the monitoring indicator. We used the HBA method to fine-tune the three parameters in the monitoring indicator proposed in this paper. These refined parameters were then used to create the monitoring indicator. We conducted tests on the test dataset and, as a result, obtained the early warning time using this monitoring indicator. We then compared this warning time with the one specified in the national standard.
3. The third experiment entailed utilizing envelope spectrum analysis on the data linked to the early warning times generated by the monitoring indicator to confirm the existence of early faults.

**Figure 2:**
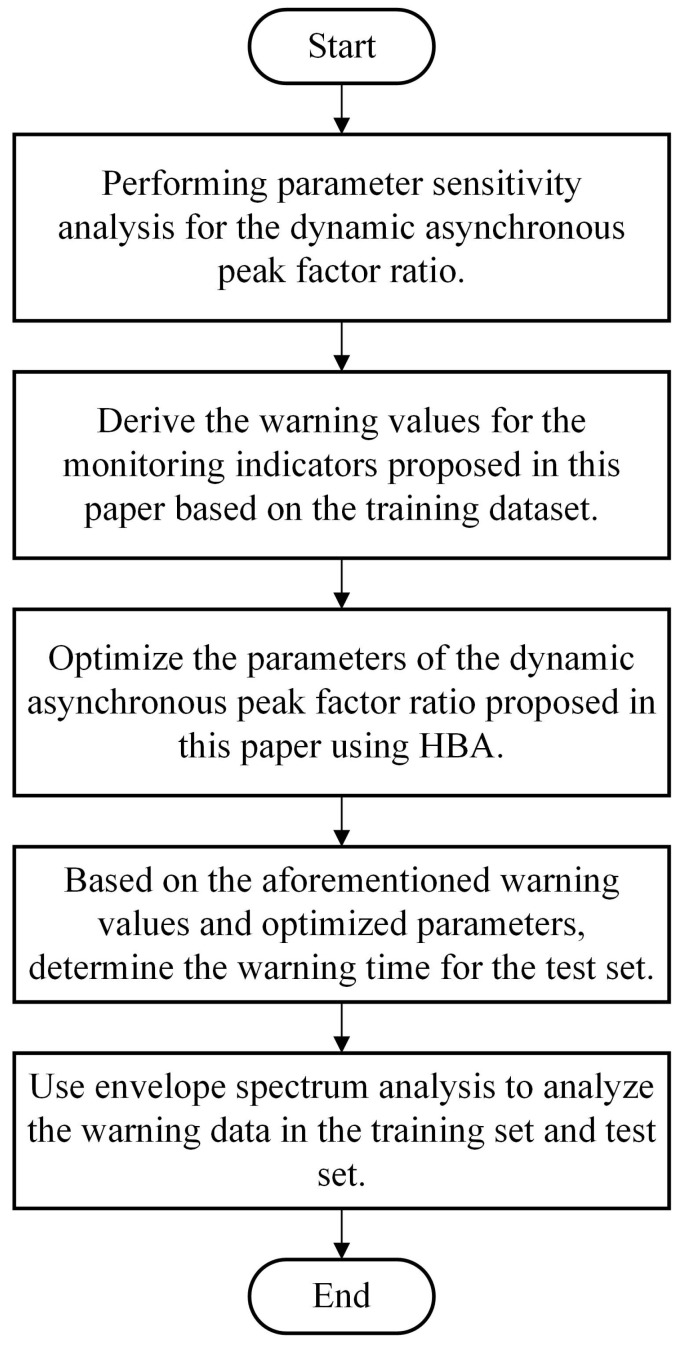
Experimental process flowchart.

In summary, the experimental process is illustrated in Figure 2.

### 3.1. Introduction to the XJTU-SY Dataset

**Figure 3:**
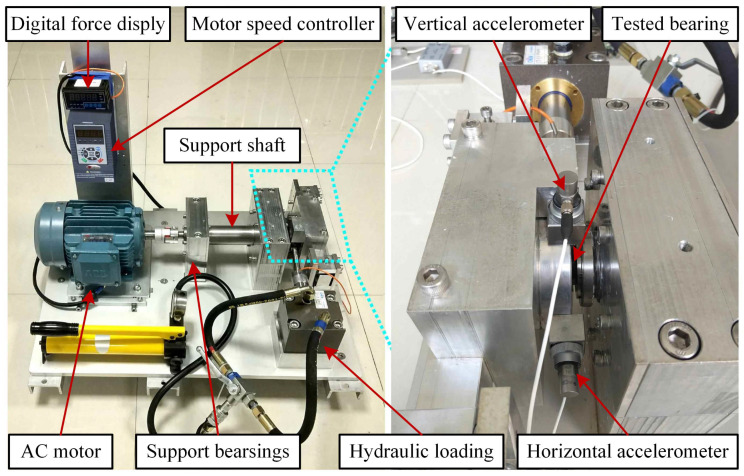
Testbed of rolling element bearings. Image source [25]. The manufacturer of the machine is Changxing Shengyang Technology Co., Ltd. located in Huzhou, China.

**Figure 4:**
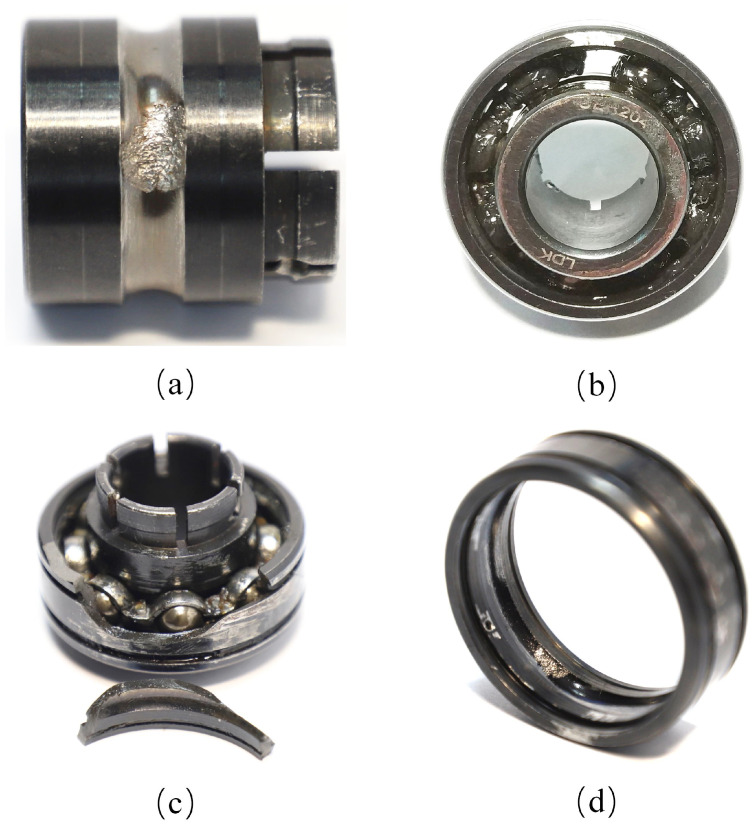
Photographs of the failure bearings. (**a**) Inner race wear. (**b**) Cage fracture. (**c**) Outer race wear. (**d**) Outer race fracture. Image source [25].

The XJTU-SY dataset was designed and collected by Professor Lei Yaguo’s team at the School of Mechanical Engineering, Xi’an Jiaotong University [25]. The test rig is shown in Figure 3, where the left side displays the overall shape and components of the test rig, and the right side provides an enlarged view of the bearing location. Acceleration sensors are placed in both the vertical and horizontal directions of the bearing, allowing data collection in two directions. The test rig consists of an AC electric motor, an electric motor speed controller, a rotating shaft, support bearings, a hydraulic loading system, and test bearings. It is capable of conducting accelerated life tests on rolling bearings under various operating conditions. During data acquisition, a sampling frequency of 25.6 kHz was used with a sampling interval of 1 min, and each sampling period lasted for 1.28 s. In each sampling period, the collected vibration signals were stored in a CSV file. The first column represents the horizontal vibration signal, while the second column represents the vertical vibration signal. The CSV files are named in chronological order of sampling times, such as 1.csv, 2.csv, …, and N.csv, where N is the total number of samples. The test bearing used in the experiments is the LDK UER204 rolling bearing. Three categories of operating conditions were designed for the experiments, as shown in Table 2, with five bearings for each condition. Table 3 provides detailed information for each test bearing, including its corresponding operating condition, the total number of data samples, the basic rated life (L10), the actual life, and the failure location. Figure 4 shows photographs of normal and faulty bearings, with failures resulting from various types of issues, including inner race wear, cage fracture, outer race wear, and outer race fracture.

The simulation in this paper was conducted in an environment with an AMD Ryzen™ 7 Mobile Processors (The manufacturer of the equipment is Lenovo Group Ltd., located in Beijing, China) with Radeon™ Graphics CPU, 2.90 GHz CPU, 32GB RAM, NVIDIA GeForce RTX 2060 GPU, and Windows 11 operating system. The programming implementation was carried out using the PyCharm 2021.3.2 (Professional Edition) software.

### 3.2. Sensitivity Analysis and Optimization of Monitoring Indicator Parameters

To validate the warning time of this model, the warning time obtained using the dynamic asynchronous peak factor ratio indicator proposed in this paper was compared with the warning time obtained from the international standard [26]. In the international standard, the monitoring indicator used is the root mean square (*RMS*) value, and the equation for the *RMS* value is as follows:

(14) $$x_{RMS}=\sqrt{\frac{1}{N}\overset{N}{\underset{n=1}{\sum }}x^{2}\left(n\right)}$$

where x(n) represents the time-domain data of the vibration signal, n=1,2,3,…,N, and *N* is the total number of samples.

**Figure 5:**
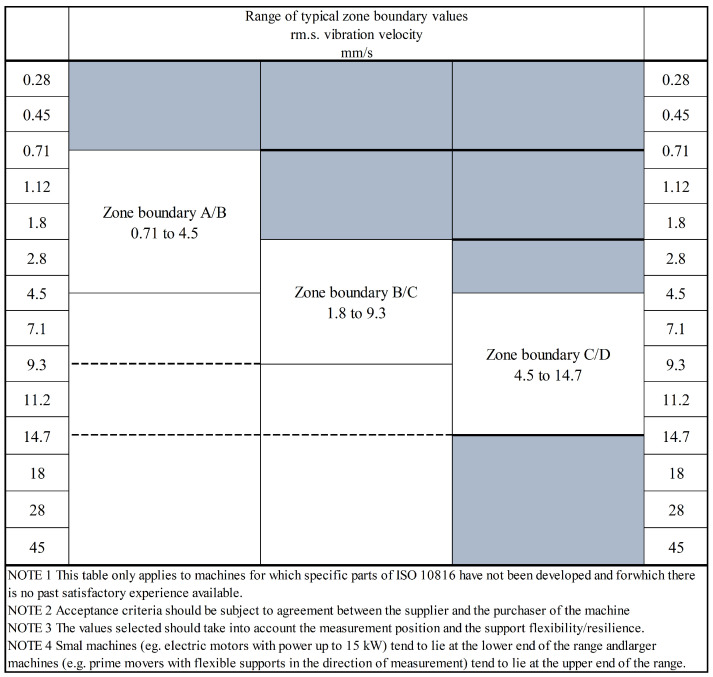
Range of typical values for the zone A/B, B/C, and C/D boundaries. Image source [26].

The informative guidelines for setting the warning value of the RMS are illustrated in Figure 5. In this figure, Region A represents the vibration level of machines in new delivery and is considered suitable for unrestricted long-term operation. Region B indicates machines with vibration levels that are generally safe for extended operation. Region C signifies machines with vibration levels that are not suitable for prolonged continuous operation. Region D corresponds to machines with high vibration levels, which are likely to cause machine damage. In this paper, we primarily focus on studying early bearing faults and issuing warnings for them. So, following the informative guidelines, small machines (e.g., electric motors with power up to 15 kW) tend to lie at the lower end of the range, and larger machines (e.g., prime movers with flexible supports in the direction of measurement) tend to lie at the upper end of the range. When the warning value is set in region C, it means that the bearing already has significant faults. In this paper, our experimental platform is designed for small-scale machines. Therefore, the RMS warning value should be positioned closer to the lower end of region B. Consequently, the RMS warning value is set to $Y_{r}$ = 2.8 mm/s.

We divided the XJTU-SY bearing dataset into training and testing sets. Since there were more data with outer ring faults in the dataset, we chose the outer ring fault data for training and testing. The bearing data used in this paper consist of horizontal vibration acceleration data. From each operational condition, one bearing data wet with an outer ring fault was selected as the testing set. Using the previously mentioned RMS warning values, we obtained the warning times for outer ring fault data in different operational conditions, as shown in Table 4.

**Figure 6:**
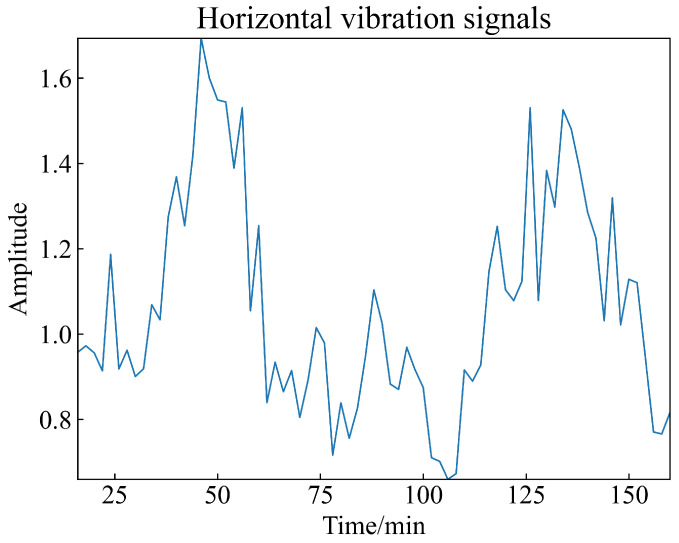
Dynamic asynchronous peak factor ratio plot for Bearing 1_2.

To obtain the warning time for this model and compare it with the warning time based on the international standard root mean square (RMS) warning values, it is necessary to set the warning values for the dynamic asynchronous peak factor ratio indicator in the monitoring metric. Here, the three main parameters that influence the monitoring indicator in this method are set to their minimum values (see Table 1 for the parameters of the dynamic asynchronous peak factor ratio), resulting in $T_{s}$, $T_{o}$, and $T_{l}$. Subsequently, graphs are generated for the training dataset Bearing 1_2, as shown in Figure 6. From Figure 6, it can be observed that Bearing 1_2 experiences its first peak at around 50 min, with this peak magnitude significantly exceeding 1.6, and the duration of the peak is approximately 20 min. As explained earlier regarding the significance of constructing the dynamic asynchronous peak factor ratio indicator, this indicator can suppress peaks caused by occasional impacts. Therefore, the duration of this peak is not expected to be very long, and the peak value is not expected to be very high. Consequently, in the period between 40 and 60 min, the rolling bearing experiences a prolonged period of impact, indicating the possibility of an early fault condition inside the bearing. Hence, the warning value is set to $Y_{c}=1.25$.

#### 3.2.1. Parameter Sensitivity Analysis

Based on the warning values set by the monitoring indicator in this paper, sensitivity analysis of the parameters is conducted using the training dataset Bearing 1_2. In this analysis, the monitoring indicator proposed in this paper has three parameters that need to be optimized.

**Figure 7:**
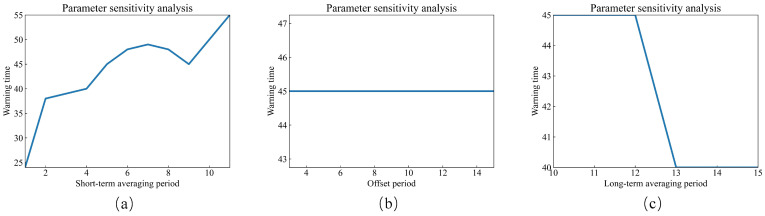
Sensitivity analysis of monitoring indicators. (**a**) Short-term averaging period. (**b**) Offset period. (**c**) Long-term averaging period.

Therefore, we keep two of these parameters fixed while allowing the third one to vary. The values of the parameters need to be integers, not decimals. The fixed parameters are chosen to be in the middle of the parameter range specified in Table 1, which means that if all three parameters are fixed, they are set as $T_{s}=5$, $T_{o}=8$, and $T_{l}=12$. The results of the sensitivity analysis are represented in terms of warning times, meaning that when different parameters are inputted, a dynamic asynchronous peak factor ratio is constructed in the training dataset. When the value of the monitoring indicator exceeds $Y_{c}=1.25$, the corresponding time is considered as the warning time for this indicator. The sensitivity analysis results for the monitoring indicator parameters in this paper are shown in Figure 7, and the sensitivity analysis data for the short-term averaging period parameters can be found in Table 5. As for the long-term averaging period parameters, their sensitivity analysis data are presented in Table 6. The warning times for the sensitivity analysis of the offset period parameter are consistently 45 min and are not presented in a separate table.

In conclusion, the following observations can be made.

1. As the short-term averaging period increases, the warning time of the monitoring indicator in this paper tends to increase. In this case, the short-term averaging period is the numerator of the monitoring indicator, indicating that when the short-term averaging period is small, it does not effectively reduce short-term peaks. This means that it cannot suppress artificial interference or occasional impacts, resulting in an increase in the monitoring indicator value and earlier warning times. When the short-term averaging period increases, the warning time is delayed, and the value of the monitoring indicator decreases. This suggests that the suppression of short-term peaks is enhanced, leading to a strengthening of long-term peaks, which corresponds to enhanced early fault detection.
2. As the offset period increases, there is no significant trend in the change in warning times for the monitoring indicator proposed in this paper. Therefore, the impact of the offset period on the monitoring indicator is relatively small.
3. With an increase in the long-term averaging period, the warning time of the monitoring indicator in this paper tends to decrease. In this case, the long-term averaging period is the denominator of the monitoring indicator. When other parameters remain constant, the warning time is advanced, indicating that the value of the monitoring indicator has increased, leading to a decrease in the denominator. When the long-term averaging period increases, it contains more early fault information in the denominator, which further causes an increase in the denominator of the peak factor. The result is an earlier warning time.
4. Sensitivity analysis reveals that the parameters with the most significant impact on the monitoring indicator are the short-term averaging period and the long-term averaging period, while the offset period has a relatively minor effect.

#### 3.2.2. HBA Parameter Optimization and Result Analysis

The tuning of parameters can be a cumbersome task, so we utilize HBA for adaptive tuning of the monitoring indicator. The population size and the maximum number of iterations are set as follows: N=50, $t_{\max}=200$. The fitness function for the HBA optimization of parameters ($T_{s}$, $T_{o}$, and $T_{l}$) is defined as follows:

(15) $$z_{f}=\left|\frac{x-\mu }{\sigma }\right|+y_{w}$$

where *x* is the first warning time when the dynamic asynchronous peak factor ratio exceeds the warning threshold. μ is the average warning time for values greater than the warning threshold. σ is the standard deviation of the warning times for values greater than the warning threshold. $y_{w}$ is the value of the dynamic asynchronous peak factor ratio for the first occurrence greater than the warning threshold.

**Figure 8:**
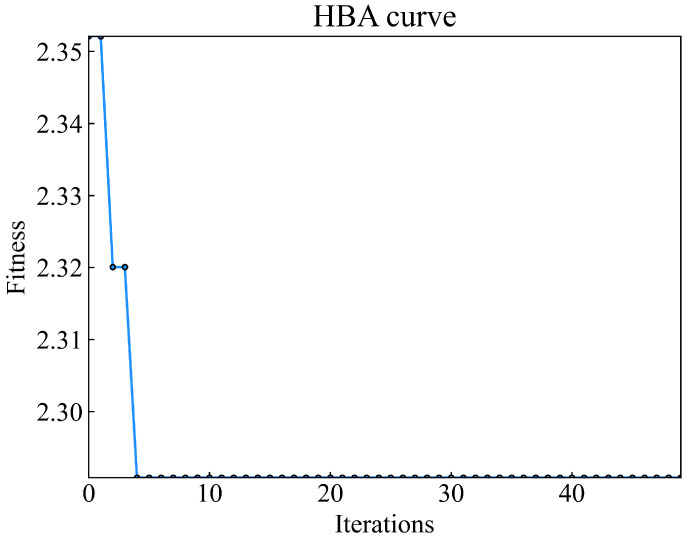
HBA fitness curve.

Furthermore, HBA is employed to optimize the parameters of the dynamic asynchronous peak factor ratio, and the fitness curve obtained on the training dataset is depicted in Figure 8. HBA escapes from local optima and achieves the best fitness in the fifth generation. The optimization time for HBA to enhance the parameters of the dynamic asynchronous peak factor ratio monitoring indicator is consistently within 0.2 s, demonstrating that HBA can effectively address the problem of finding the optimal solution. It excels in various aspects, including algorithm structure, convergence speed, and the balance between exploration and exploitation.

The optimal honey badger position found during the search was [3,15,13], indicating the optimal parameters for optimizing the monitoring indicator of the dynamic asynchronous peak factor ratio are $T_{s}=3$, $T_{o}=15$, and $T_{l}=13$.

**Figure 9:**
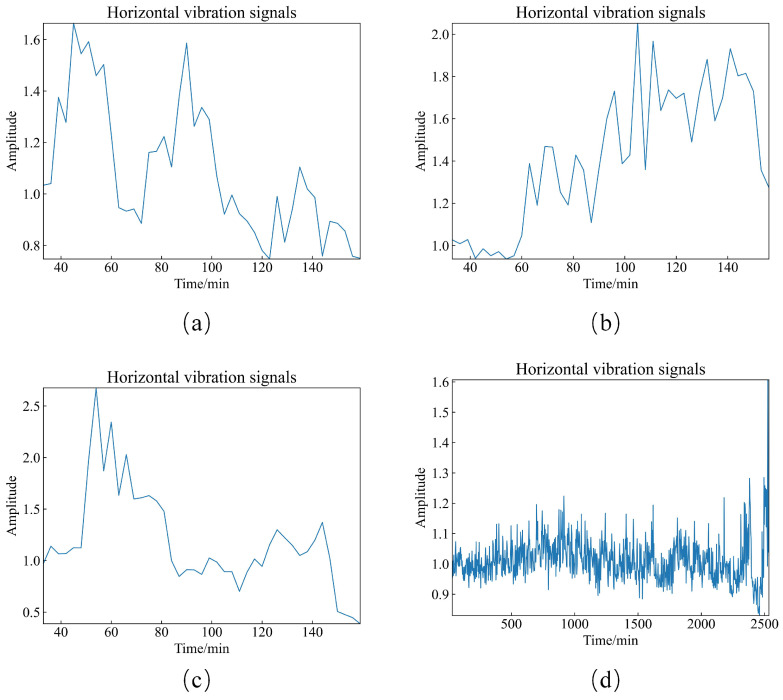
Dynamic Asynchronous Peak Factor Ratio plots. (**a**) Bearing 1_2. (**b**) Bearing 1_3. (**c**) Bearing 2_2. (**d**) Bearing 3_1.

Based on the optimization performed using HBA for the monitoring indicator for the dynamic asynchronous peak factor ratio, the optimized short, offset, and long periods are obtained. Finally, the monitoring indicator for the optimized dynamic asynchronous peak factor ratio is constructed. The optimized monitoring indicator is then tested on the test dataset, and graphs for the dynamic asynchronous peak factor ratio are plotted accordingly, as shown in Figure 9. In the training dataset Bearing 1_2, the warning threshold for the monitoring indicator of the dynamic asynchronous peak factor ratio was set. During testing in the test dataset, the time at which the first value of the dynamic asynchronous peak factor ratio exceeds the warning threshold is considered the warning time obtained using this method. Finally, the warning time obtained in this paper is compared with the warning time obtained in the international standard. This comparison allows us to determine how much earlier the warning time in this paper is compared to that obtained using the traditional method. The warning times for both the proposed method and the traditional method in the training and test datasets, along with the lead time, are summarized in Table 7.

From the preceding text and Table 7, it is evident that the construction process of the dynamic asynchronous peak factor ratio proposed in this paper is not complicated. Compared to international standards, the monitoring indicator introduced in this paper offers early warning times that are advanced by at least 33 min. Experimental results affirm the significant effectiveness of the monitoring indicator proposed in this paper for early fault warnings in rolling bearings.

### 3.3. Early Fault Verification

Although envelope spectrum analysis effectively demodulates vibration signals for fault detection and diagnosis, this method lacks real-time fault-monitoring capabilities. In practice, it is common to perform envelope spectrum analysis on data collected after a national standard warning to determine the type of bearing fault. The metric proposed in this paper allows for an early warning time of half an hour before the national standard. Additionally, envelope spectrum analysis is applied to the bearing data obtained after the warning based on the Dynamic Asynchronous Peak Factor Ratio to verify the presence of early-stage faults. Here, Equation (13) is used to calculate the characteristic frequencies of outer ring faults for three operating conditions in the dataset, as shown in Table 8.

**Figure 10:**
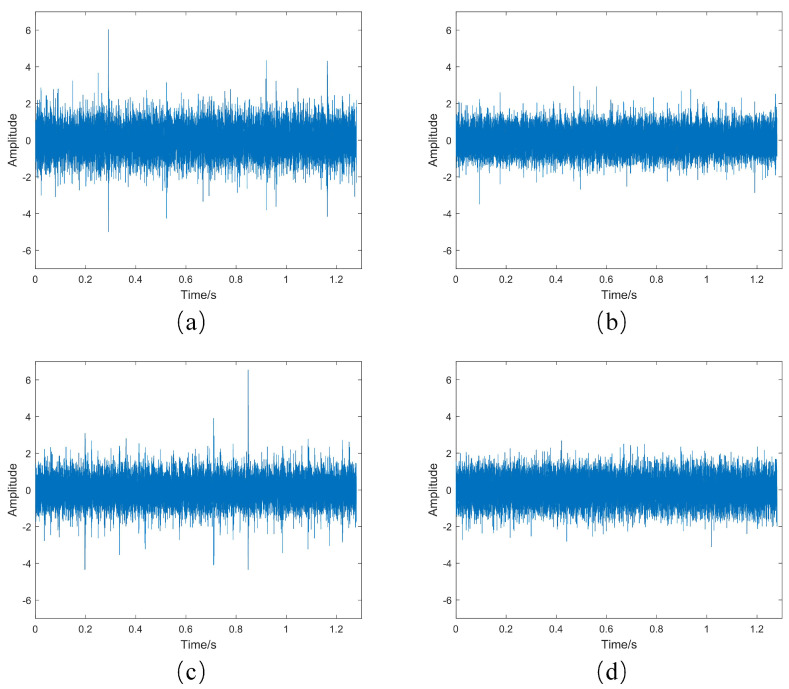
Original vibration signal waveforms. (**a**) Bearing 1_2—39.csv. (**b**) Bearing 1_3—63.csv. (**c**) Bearing 2_2—51.csv. (**d**) Bearing 3_1—2382.csv.

**Figure 11:**
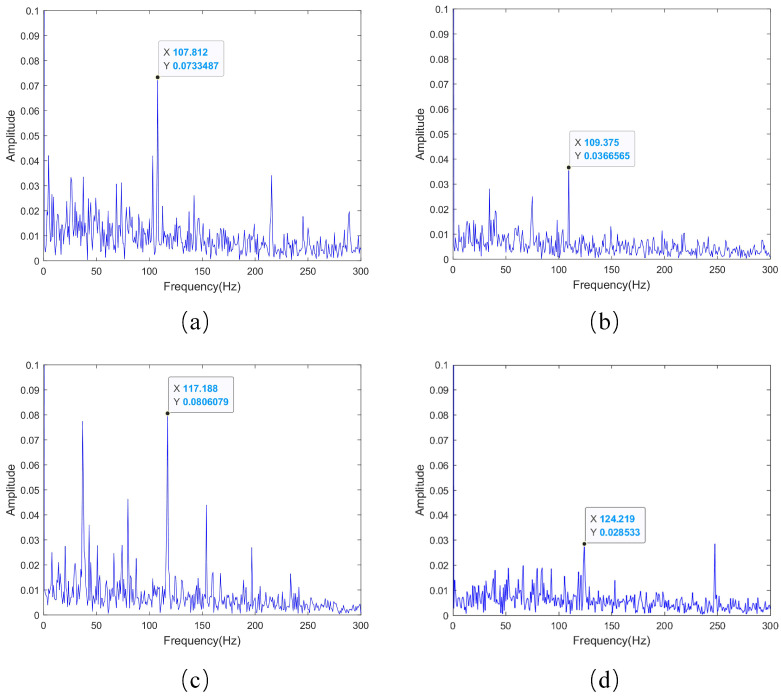
Envelope spectrum plots. (**a**) Bearing 1_2—39.csv. (**b**) Bearing 1_3—63.csv. (**c**) Bearing 2_2—51.csv. (**d**) Bearing 3_1—2382.csv.

Based on the warning sample data obtained from the monitoring indicators proposed in this paper, i.e., files such as Bearing 1_2—39.csv, Bearing 1_3—63.csv, Bearing 2_2—51.csv, and Bearing 3_1—2382.csv, the original vibration signal waveforms and envelope spectra were plotted separately, as shown in Figure 10 and Figure 11.

An analysis of the original vibration signal waveforms for the four warning samples shows that Bearing 1_2—39.csv and Bearing 2_2—51.csv exhibit significant amplitude spikes. These are the data that the monitoring indicator proposed in this paper warned about, indicating the impact caused by early bearing failure. On the other hand, Bearing 1_3—63.csv and Bearing 3_1—2382.csv do not show obvious spikes in the original vibration signal waveforms but exhibit a clear deteriorating trend. The next step involves the envelope spectrum analysis of these samples.

Based on the envelope spectrum plots in Figure 11, and observing the envelope spectrum plots in each of the early outer race fault samples, there are clear indications of the characteristic frequencies of the outer race fault. For example, in Bearing 1_2—39.csv, the outer race fault’s characteristic frequency is 107.812 Hz, while the calculated ideal outer race fault’s characteristic frequency is 107.907 Hz. The small difference between the two suggests that the bearing is experiencing an early-stage outer race fault. As for Bearing 1_3—63.csv, Bearing 2_2—51.csv, and Bearing 3_1—2382.csv, their outer race fault’s characteristic frequencies differ significantly from the ideal ones. This is because early-stage fault signals in bearings are weak and easily susceptible to interference from other signals, causing the fault characteristic frequencies to fluctuate near the ideal values. Through envelope spectrum analysis of the warning samples, it is evident that in each sample, the early-stage outer race fault in the bearings is prominently detected, thereby demonstrating that the method proposed in this paper is capable of timely and accurate early-stage fault detection in bearings.

## 4. Conclusions

To ensure timely early fault warnings for rolling bearings, this paper introduces a rolling bearing state monitoring method based on the dynamic asynchronous peak factor ratio. When comparing this method with the warning values established by international standards, the results indicate that HBA can optimize key parameters in the model for early fault monitoring for rolling bearings. For the XJTU-SY dataset, the warning times obtained from the model developed in this paper precede those of the international standards by 24 min, 33 min, 86 min, and 135 min, respectively. Furthermore, the monitoring indicator created in this study can provide valuable technical support for early fault condition monitoring in rolling bearings.

In future research, it will be essential to take into account the potential consequences of severe faults in rolling bearings. While this method demonstrates proficiency in accurately monitoring early-stage faults, the stability of the Dynamic Asynchronous Peak Factor Ratio index may wane when severe faults occur in rolling bearings. Therefore, future research should prioritize addressing severe faults in rolling bearings.

## Figures and Tables

**Table 1:** Parameter ranges for the dynamic asynchronous peak factor ratio.

Parameter	Value
$T_{s}$	[2,8]
$T_{o}$	[3,15]
$T_{l}$	[10,15]

**Table 2:** Operating condition.

Operating Condition	Condition 1	Condition 2	Condition 3
Rotational speed (r/min)	2100	2250	2400
Radial load (kN)	12	11	10

**Table 3:** XJTU-SY Bearing Datasets.

Operating Condition	Bearing Dataset	Number of Files	Bearing Lifetime	Fault Element
Condition 1 (35 Hz/12 kN)	Bearing 1_1	123	2 h 3 min	Outer race
	Bearing 1_2	161	2 h 41 min	Outer race
	Bearing 1_3	158	2 h 38 min	Outer race
	Bearing 1_4	122	2 h 2 min	Cage
	Bearing 1_5	52	52 min	Inner race and outer race
Condition 2 (37.5 Hz/11 kN)	Bearing 2_1	491	8 h 11 min	Inner race
	Bearing 2_2	161	2 h 41 min	Outer race
	Bearing 2_3	533	8 h 53 min	Cage
	Bearing 2_4	42	42 min	Outer race
	Bearing 2_5	339	5 h 39 min	Outer race
Condition 3 (40 Hz/10 kN)	Bearing 3_1	2538	42 h 18 min	Outer race
	Bearing 3_2	2496	41 h 36 min	Inner race, ball, cgar and outer race
	Bearing 3_3	371	6 h 11 min	Inner race
	Bearing 3_4	1515	25 h 15 min	Inner race
	Bearing 3_5	114	1 h 54 min	Outer race

**Table 4:** Division of the XJTU-SY bearing datasets and international standard warning times.

Dataset	Bearing	$Y_{r}$ Warning Times/Min
Training Set	Bearing 1_2	63
Testing set	Bearing 1_3	149
	Bearing 2_2	84
	Bearing 3_1	2517

**Table 5:** Sensitivity analysis of the short-term averaging period.

Short-Term Averaging Period	Offset Period	Long-Term Averaging Period	Warning Times/Min
2			38
3			39
4			40
5	8	12	45
6			48
7			49
8			48

**Table 6:** Sensitivity analysis of the long-term averaging period.

Short-Term Averaging Period	Offset Period	Long-Term Averaging Period	Warning Times/Min
5	8	10	45
		11	45
		12	45
		13	40
		14	40
		15	40

**Table 7:** Overview of the warning times for the XJTU-SY dataset.

Dataset	Bearing	$Y_{r}$ Warning Times/Min	$Y_{c}$ Warning Times/Min	Lead Times/Min
Training set	Bearing 1_2	63	39	24
Testing set	Bearing 1_3	149	63	86
	Bearing 2_2	84	51	33
	Bearing 3_1	2517	2382	135

**Table 8:** Characteristic Frequencies.

Fault Types	Condition 1	Condition 2	Condition 3
Outer Race Fault Feature Frequencies	107.907 Hz	115.615 Hz	123.323 Hz

## Data Availability

This study used the XJTU-SY bearing public data set. The data can be found here: https://biawang.tech/xjtu-sy-bearing-datasets (accessed on 28 September 2023).
